# Supplementary material for: Diagnostic Accuracy of a Portable ECG Device in Rowing Athletes
Source: Diagnostics (Basel). 2022 Sep 20;12(10):2271. doi: 10.3390/diagnostics12102271 (PMC9600971; doi:10.3390/diagnostics12102271)
Supplement: Supplementary file 1 [file diagnostics-12-02271-s001.zip › diagnostics-1894098-supplementary .pdf]

diagnostics

MDPI

Supplementary file

1: Experimental protocol for Phase I

Experimental protocol

Participants attended the Human Performance Laboratory of the hospital for exercise testing on two occasions, separated by 48 hours. Participants were asked to refrain from ingesting caffeine and alcohol, and to avoid exercise or strenuous physical activity for 24 hours prior to each visit. During each visit, participants were fitted with the PluxECG ~~device~~ system and a reference 12- lead ECG system; MGC Diagnostics, the gold standard for exercise ECG testing (http://www.mgcdiagnostics.com). A laboratory technician trained in ECG testing fitted participants with the devices during each visit. Prior to at-tachment of the devices, the chest region of participants was shaved, cleaned, abrased and dried. The PluxECG device electrodes were positioned on the right sternum, left sternum, and mid-axillary line. The MGC Diagnostics system electrodes were attached according to standard 12-lead configuration. 3m Red Dot 2238 electrodes were employed for both the PluxECG and MGC Diagnostics systems.

The exercise test was divided into two different phases: Step Protocol and Ramp Pro-tocol. Figure S1 displays an overview of the activities involved in each of the protocols, which include rest and exercise at different levels of rowing intensity to maximise HR.

Prior to commencing both exercise protocols, participants were required to complete a 5-minute warm up on a Concept2 (Model D, USA) rowing ergometer at a power output equivalent to the first stage of the subsequent test. The Step Protocol required participants to perform five × two-minute incremental rowing phases. Each of the five stages were separated by a one-minute recovery, during which participants blood lactate was meas-ured by taking a blood sample from the earlobe via Lactate Plus (Nova Biomedical, Wal-tham, MA) (see Figure S1a). Participants starting workload and incremental load were de-termined during their pre-assessment using their previous best 2000m row time. Subse-quently, rowers started at a load ranging between 140 and 200 W and an incremental in-crease of 15–45 W. Table S1 provides an example of experimenter's notes for one participant during the Step Protocol.

Following a period of rest of 60 minutes, participants underwent the second phase of exercise testing following the Ramp Protocol (see Figure S1b). This required participants to perform five x two-minute exercise stages at specific stroke rates, proceeded by a period of rest. At the end of each two-minute exercise stage, participants were instructed to row at a higher stroke rate.

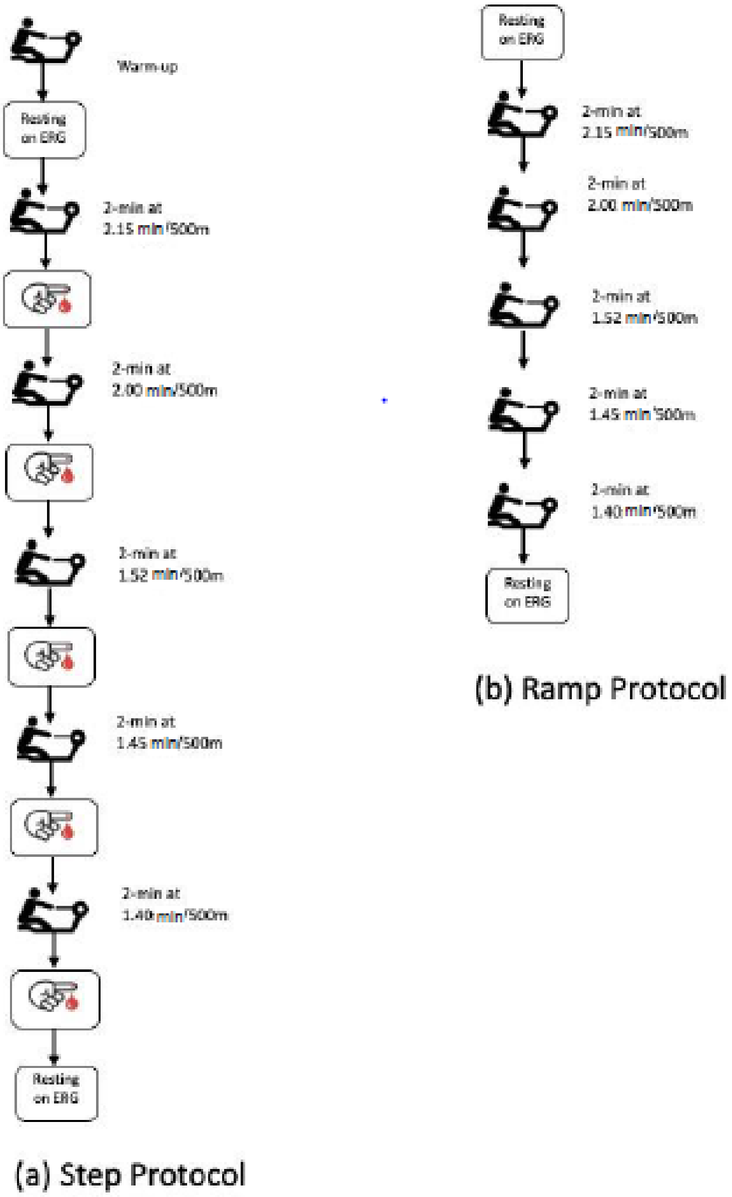

Figure S1. (a) Step Protocol with blood sample taken after each 2-minute stage, (b) Ramp Protocol progressing to a higher stroke rate every 2-minutes.

2: Data Processing and Synchronisation of data.

PluxECG device

ECG Data recorded from the PluxECG ~~device~~ sensor was analysed using customised scripts developed using MATLAB R2020a [MathWorks - www.mathworks.com]. The **Figure S2** below displays the series of processing steps involved in the extraction of HR data from the PluxECG. ECG data was acquired by the PluxECG device at a sampling rate of 1000Hz. Initially, data was filtered using a zero phase 3rd order Butterworth bandpass filter with cut-off frequencies of 5Hz and 15Hz. The signal was subsequently differenti-ated in order to emphasise the QRS complex and a derivating lowpass filter was em-ployed. This filter design is a maximally flat low differentiator, that behaves as an ideal differentiator up to one tenth of the Nyquist frequency; 50Hz (21). The implementation of the bandpass filter before the derivating filter ensured that the data contained frequencies sufficiently below this limit. Next, the data was squared to further emphasise areas where rate of change was greatest in the signal. Finally, the signal was averaged using a window size of 0.150s (150 samples) to remove any remaining high frequency noise. The Pan Tompkins QRS algorithm was then implemented to extract R-R intervals, which were sub-sequently converted into instantaneous HR. As HR data was computed on an instantane-ous basis for each R-R interval, a median filter using a window size of 5 samples was applied to remove any high frequency low amplitude noise present.

MGC Diagnostics System

Time stamped HR data from the MGC Diagnostics system was stored in a unique logfile (.txt) for each test and each participant. The 12-lead ECGs were interpreted by a board-certified cardiologist for abnormalities. A custom MATLAB script was created to extract HR data and corresponding time stamp data from the unique logfile for each test protocol and each participant. Extracted data was then stored separately for subsequent analysis.

Synchronisation of data

Although data from the PluxECG and MGC Diagnostics devices systems were obtained contemporaneously, no hardwired synchronisation option was available. However, based on the protocols applied, temporal synchronisation was deemed possible. By noting the start and end timestamps for each exercise protocol reported in the systems logfile, then calculating the number of HR measurements ensuing, the MGC Diagnostic system sampling rate was calculated as 0.5938Hz. Subsequently, comparative analysis was carried out for each 2-minute exercise stage for each participant for the Step Protocol and Ramp Protocol.

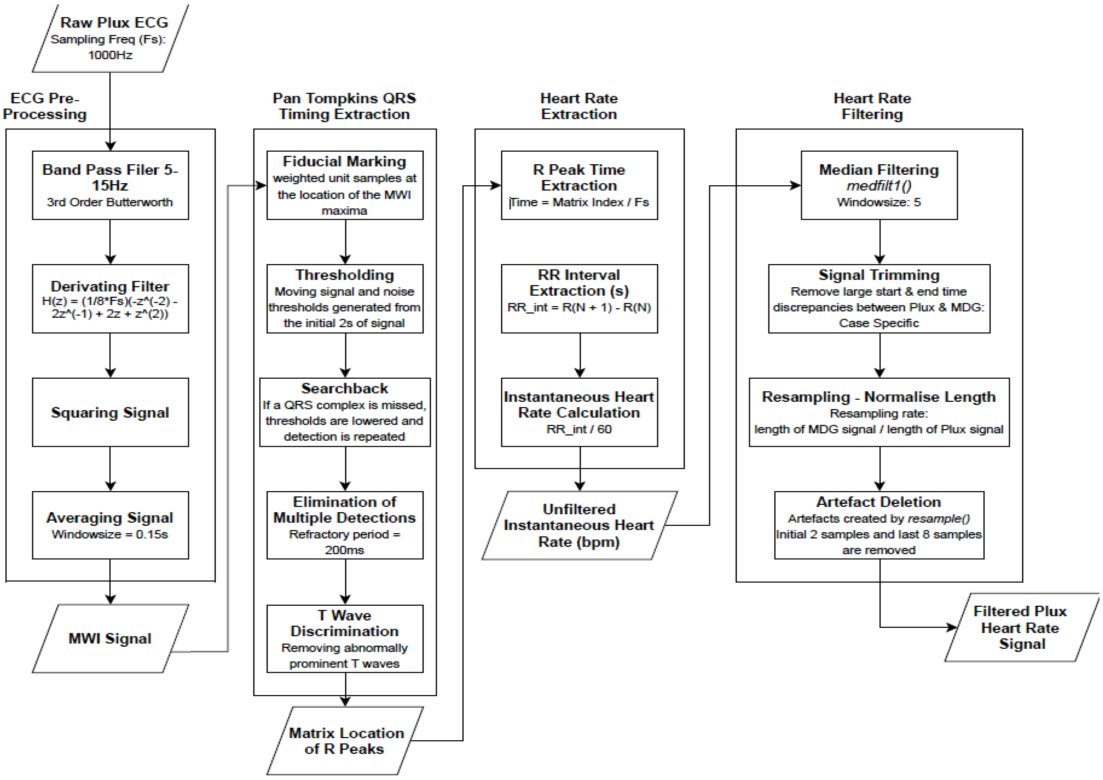

Figure S2. Processing steps to extract HR date from the PluxECG system.

3: Re-sampling method allowing the lab-based 12-lead ECG assessment and Plux-ECG signals to contain the same number of samples.

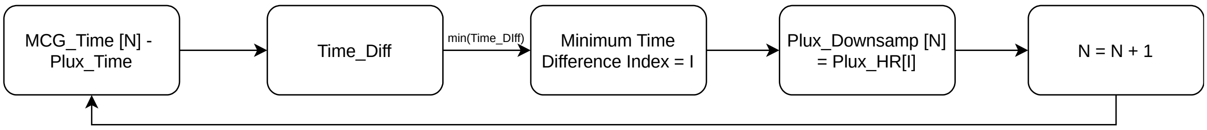

Figure S3. Re-sampling method allowing the lab-based 12-lead ECG assessment and PluxECG signals to contain the same number of samples.

diagnostics

MDPI

4: Elevated heart rate correlation and covariance.

Table S1. Elevated heart rate correlation and covariance.

**Participant**	**1**		**2**		**3**	**4**	**5**	
**Day**	Tues	Tues	Tues	Tues	Tues	Thurs	Thurs	Thurs
**Test**	Step	Ramp	Step	Ramp	Step	Step	Step	Ramp
**Threshold**	120	120	130	120	130	130	130	130
**Covariance**	224.648	174.816	418.523	175.780	406.781	438.218	260.349	326.430
**Correlation**	0.9909	0.9585	0.9953	0.9762	0.9962	0.9980	0.8474	0.9781

5: Heart rate time series data calculated from the PluxECG and lab-based 12-lead ECG assessment from one subject undergoing the Step Protocol.

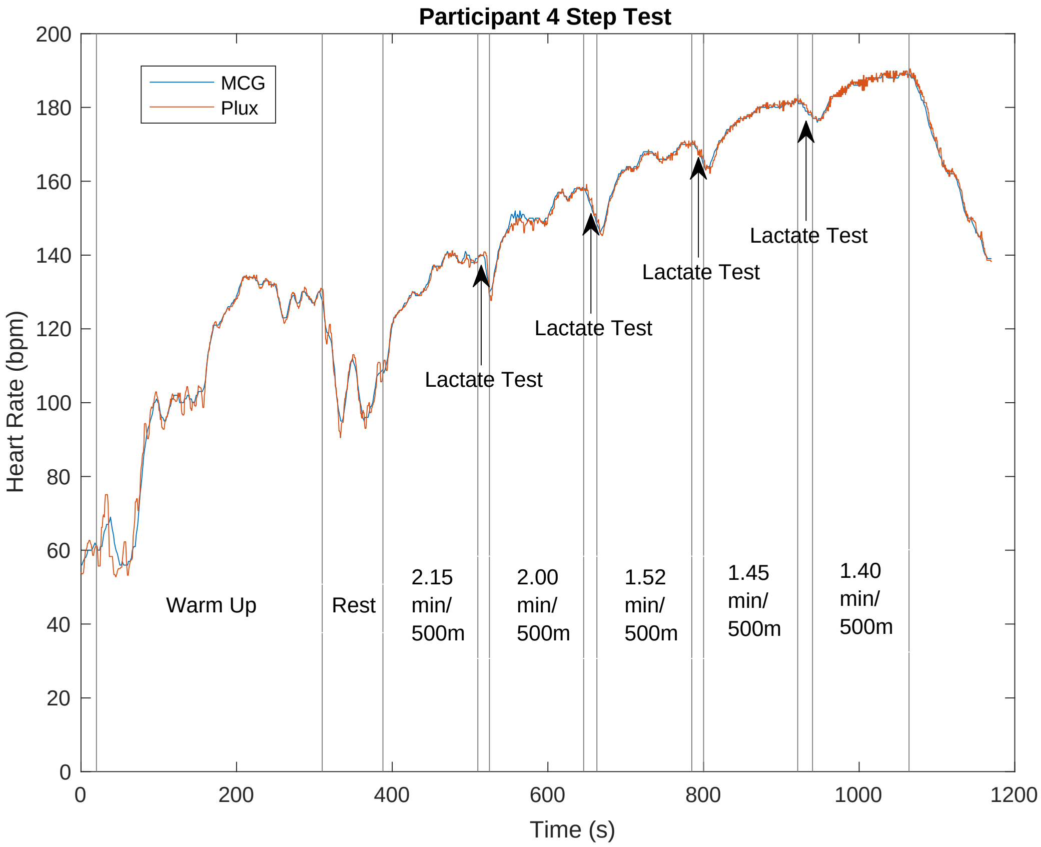

Figure S4. Heart rate time series data calculated from the PluxECG lab-based 12-lead ECG assessment from one subject undergoing the Step Protocol. Note alignment of data from both systems aligned to the start of the test.

6: Heart rate time series data calculated from the PluxECG and lab-based 12-lead ECG assessment from one subject undergoing the Ramp Protocol.

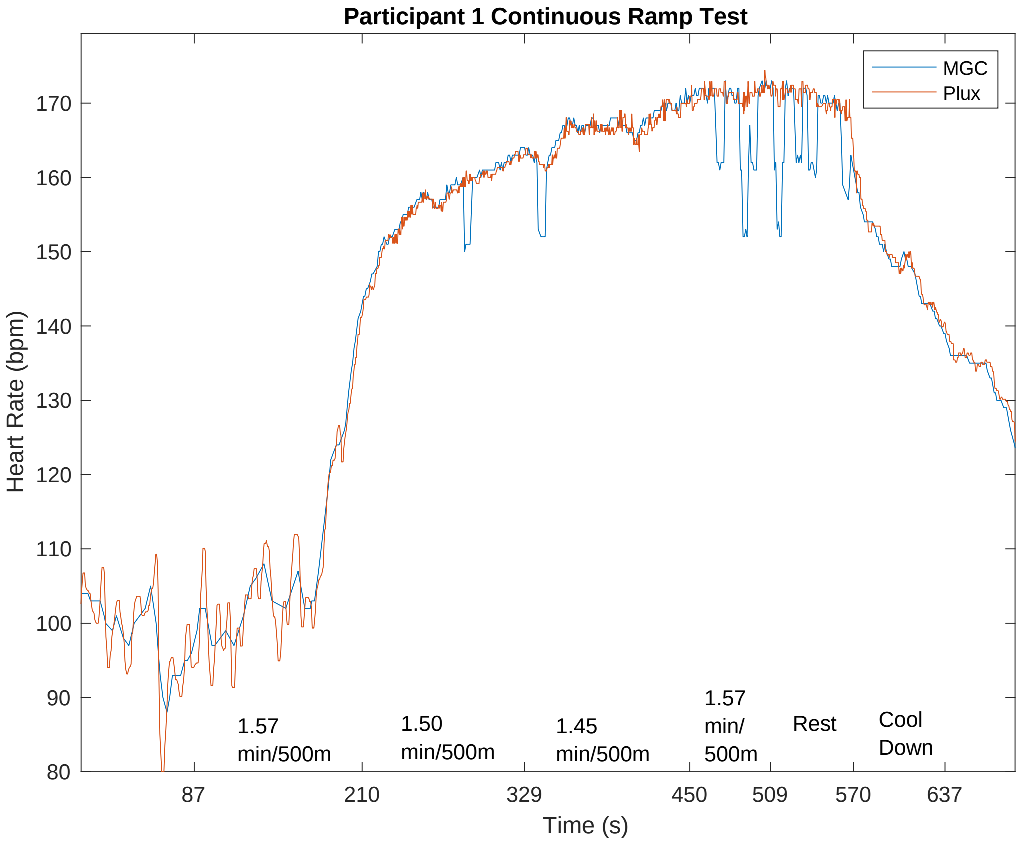

Figure S5. Heart rate time series data for Participant 1 calculated from the PluxECG lab-based 12-lead ECG assessment from one subject undergoing the Ramp Protocol. Note alignment of data from both systems aligned to the start of the test.

7: Movement artifact in heart rate time series.

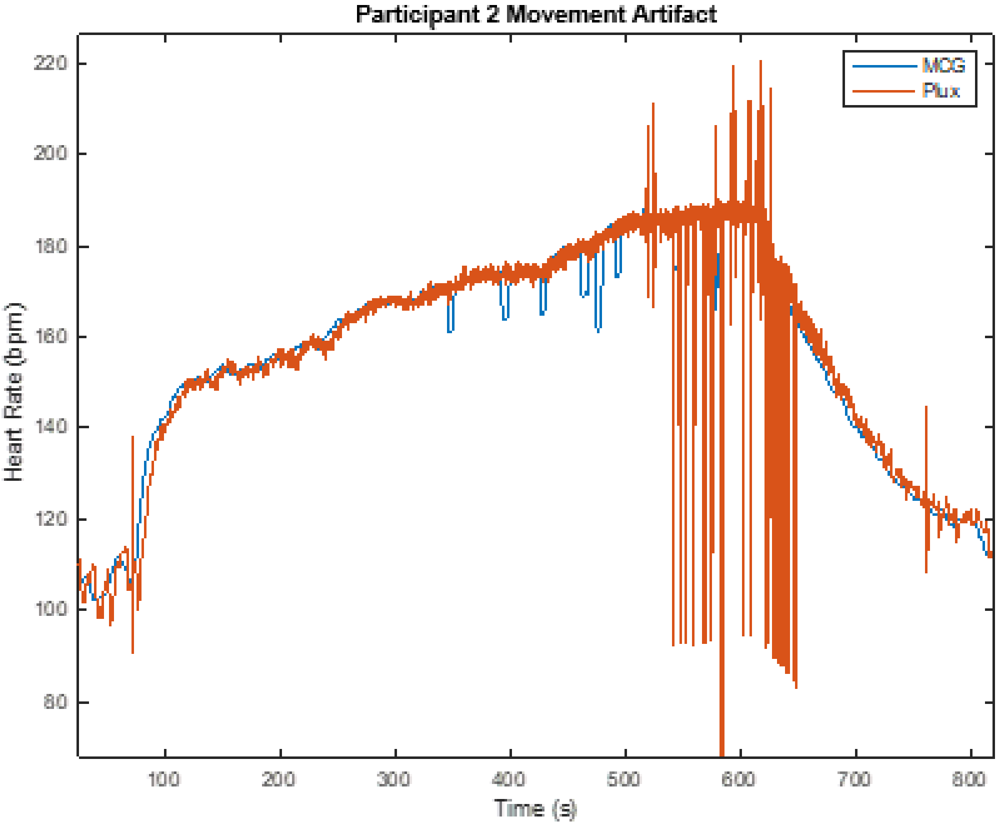

Figure S6. Example of movement artifact in heart rate time series data calculated from the PluxECG and lab-based 12-lead ECG assessment from one subject undergoing the Ramp Protocol.
